# The Pangenome of Gram-Negative Environmental Bacteria Hides a Promising Biotechnological Potential

**DOI:** 10.3390/microorganisms11102445

**Published:** 2023-09-29

**Authors:** Cláudia Covas, Gonçalo Figueiredo, Margarida Gomes, Tiago Santos, Sónia Mendo, Tânia S. Caetano

**Affiliations:** CESAM and Department of Biology, University of Aveiro, 3810-193 Aveiro, Portugal; claudia.covas@ua.pt (C.C.); henriques.figueiredo@ua.pt (G.F.); smendo@ua.pt (S.M.)

**Keywords:** specialized metabolites, siderophore, carotenoids, squalene, agriculture, PGPB

## Abstract

Secondary metabolites (SMs) from environmental bacteria offer viable solutions for various health and environmental challenges. Researchers are employing advanced bioinformatic tools to investigate less-explored microorganisms and unearth novel bioactive compounds. In this research area, our understanding of SMs from environmental Gram-negative bacteria lags behind that of its Gram-positive counterparts. In this regard, *Pedobacter* spp. have recently gained attention, not only for their role as plant growth promoters but also for their potential in producing antimicrobials. This study focuses on the genomic analysis of *Pedobacter* spp. to unveil the diversity of the SMs encoded in their genomes. Among the 41 genomes analyzed, a total of 233 biosynthetic gene clusters (BGCs) were identified, revealing the potential for the production of diverse SMs, including RiPPs (27%), terpenes (22%), hybrid SMs (17%), PKs (12%), NRPs (9%) and siderophores (6%). Overall, BGC distribution did not correlate with phylogenetic lineage and most of the BGCs showed no significant hits in the MIBiG database, emphasizing the uniqueness of the compounds that *Pedobacter* spp. can produce. Of all the species examined, *P. cryoconitis* and *P. lusitanus* stood out for having the highest number and diversity of BGCs. Focusing on their applicability and ecological functions, we investigated in greater detail the BGCs responsible for siderophore and terpenoid production in these species and their relatives. Our findings suggest that *P. cryoconitis* and *P. lusitanus* have the potential to produce novel mixtures of siderophores, involving bifunctional IucAC/AcD NIS synthetases, as well as carotenoids and squalene. This study highlights the biotechnological potential of *Pedobacter* spp. in medicine, agriculture and other industries, emphasizing the need for a continued exploration of its SMs and their applications.

## 1. Introduction

Environmental natural products produced by microorganisms are and have been the most promising source for the discovery of new drugs [1,2]. The interest in exploring less-studied microorganisms for the production of novel molecules with relevant bioactivities has gained special importance in recent years, benefiting from the new bioinformatic tools and “omics” technologies available. Genome mining approaches, for instance, have been a powerful tool for identifying genes involved in the production of secondary metabolites (SMs) in genomes of poorly studied bacterial groups [3,4]. Such strategies lead to the discovery of a plethora of microbial compounds with applications in medicine and are being increasingly applied to other fields such as agriculture, nutrition, cosmetics and biomaterials, among others [5,6,7,8].

A less explored group of bacteria is *Pedobacter* spp., comprising Gram-negative bacteria from the Bacteroidota phylum, often found in soils or as members of the rhizosphere [9,10]. Moreover, this genus are known as environmental superbugs because they are capable of coping with the vast majority of known antibiotics [11,12]. Some *Pedobacter* spp. display antimicrobial activity, but the characterization of their bioactive compounds is currently limited to an antifungal chitinase and nonribosomal peptides known as (iso)pedopeptins [12,13,14,15,16]. Notably, pedopeptins inhibit the growth of some antibiotic-resistant bacteria, which are classified by the WHO as a great threat to human health [17]. Other studies suggest *Pedobacter* spp. as producers of novel lanthipeptides, which are RiPPs described as having a wide variety of biological activities, including antimicrobial, antiviral and antiallodynic [18,19,20]. This highlights the potential of antimicrobials derived from *Pedobacter* spp. for diverse applications in the fields of medicine and food, including biopharmaceuticals and biopreservatives [21,22]. However, we anticipate that the biotechnological scope of this genus is considerably wider, potentially encompassing agricultural biotechnology, as certain species have been identified as plant growth-promoting bacteria (PGPB) while exhibiting the ability to suppress plant root diseases [23,24,25,26].

Recognizing the biotechnological importance of the *Pedobacter* genus, we leveraged genomic analysis tools to reveal the array of secondary metabolites (SMs) encoded in its pangenome. These include the biosynthetic gene clusters (BGCs) responsible for the synthesis of terpenes (including carotenoids), nonribosomal peptides (NRPs), polyketides (PKs), siderophores, and ribosomally synthesized and post-translationally modified peptides (RiPPs), among other compounds.

## 2. Materials and Methods

### 2.1. Identification of BGCs

For all *Pedobacter* species genomes available in the National Center for Biotechnology Information (NCBI) database, a screening for candidate biosynthetic gene clusters (BGCs) was performed using the specialized metabolite identification pipeline antiSMASH version 5.0 [27]. *Pedobacter* species genome sequences were obtained from http://www.ncbi.nlm.nih.gov/genone/?term=pedobacter, accessed on 6 June 2019. The distribution of BGCs identified via the antiSMASH analysis among the *Pedobacter* species was visualized in a circular chord diagram, generated using Circos [28]. BiG-SCAPE version 20181005 (available at https://git.wageningenur.nl/medema-group/BiG-SCAPE/-/wikis/installation; accessed on 10 February 2019) was used locally to analyze the 233 BGCs as individual .gbk files that were downloaded from the antiSMASH database (10 March 2019) [29,30]. Phylogenetic trees provided by CORASON were generated during the BiG-SCAPE analysis. A BGC distribution analysis for the *Pedobacter* genus was performed using the ClustVis (https://biit.cs.ut.ee/clustvis/) tool, which allowed for the generation of the principal components analysis (PCA) plot and heatmaps [31].

### 2.2. Phylogenetic Tree Construction

A 16S rRNA gene tree was constructed in MEGA 7 [32] using the neighbor-joining (NJ) method [33]. The model applied was the Kimura 2-parameter with gamma distribution and invariant sites (G + I) since it was the model with the lowest BIC (Bayesian Information Criterion), determined via the model prediction option of MEGA 7. The *Pedobacter* species 16S rRNA gene sequences were retrieved from the GenBank database and aligned using the CLUSTALX algorithm [34]. The confidence level of the branches was determined using the bootstrap method with 1000 replications. To obtain the NIS synthetases phylogenetic tree, the protein sequences of the characterized proteins were obtained from the Uniprot database and aligned using MUSCLE [35] with the *P. cryoconitis*, *P. lusitanus*, *P. hartonius* and *P. himalayensis* NIS synthetases. Their evolutionary history was also inferred with MEGA 7 using the maximum likelihood method. A discrete gamma distribution was used to model the evolutionary rate differences among the sites (5 categories (+G, parameter = 1.5404)). The rate variation model allowed for some sites to be evolutionarily invariable ([+I], 1.00% sites).

### 2.3. Analysis of Siderophore and Terpene BGCs

The sequence of all siderophores and terpenes BGCs analyzed via comparative genomics were retrieved directly from the antiSMASH database or taken directly from the Nucleotide database from the National Center for Biotechnology Information (NCBI) database [36]. The latter were identified following a microbial Blast analysis. The InterPro [37] and NCBI Conserved Domain [38] webtools were used to predict the protein domains and infer their functions. The putative genes involved in the synthesis of siderophores and terpenes were colored and viewed with the Artemis software tool [39]. A comparative genomics analysis was performed and the similarities between clusters were visualized with Easyfig. 2.1 with a tblastx analysis [40]. The final images were constructed with OmniGraffle Professional software 5.4.4.

## 3. Results and Discussion

### 3.1. Pedobacter spp. Encode the Production of a High Diversity of SMs

**Figure 1:**
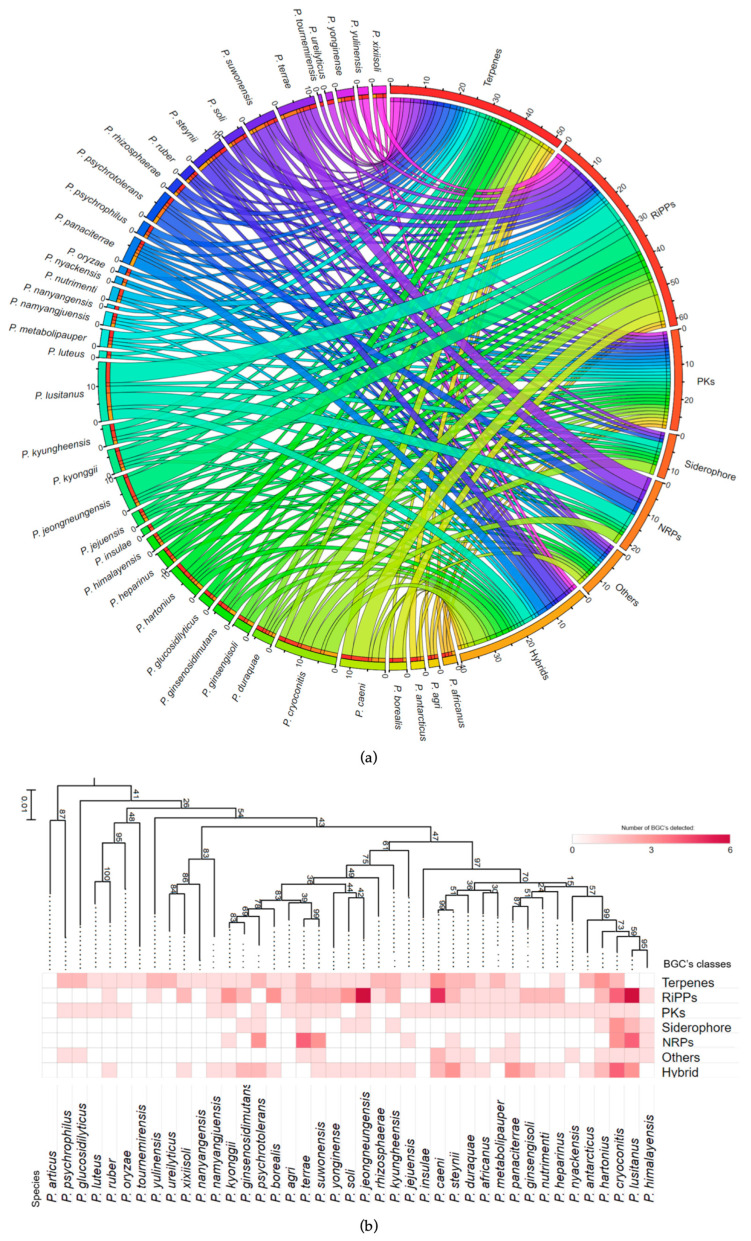
Representation of the various classes of BGCs identified in *Pedobacter* spp.; (**a**) their numbers according to the species and (**b**) according to their phylogeny and species. The neighbor-joining (NJ) tree (**b**) was based on a 16S rRNA gene sequence analysis and its scale corresponds to one nucleotide substitution per 100 nucleotides.

The potential of *Pedobacter* to produce SMs was evaluated by examining the genomes of *Pedobacter* spp. (*n* = 41) with antiSMASH [41]. This tool has gained widespread use in genome mining, allowing for the discovery of potential cryptic gene clusters that encode natural products that are not normally produced under laboratory conditions. For instance, it has been instrumental in uncovering various specialized metabolites in *Streptomyces* spp. and RiPPs in anaerobic bacteria, as well as shedding light on the unexplored metabolism of *Myxobacteria* [42,43,44]. Collectively, the genus *Pedobacter* harbored a total of 233 BGCs, with an average of 6 BGCs per genome (Figure 1a; Table S1). Most of the BGCs encode the potential to produce RiPPs (27%), followed by terpenes (22%), hybrid SMs (17%) and PKs (12%). Additionally, we identified BGCs responsible for the production of NRPs (9%), other SMs (7%) and siderophores (6%), but in lower percentages (Figure 1a; Table S1). The mean count of BGCs per genome in this study falls below the values reported in analogous research involving metabolically versatile Gram-positive bacteria, such as the genus *Rhodococcus* (mean count of 17) [45], or the Gram-negative genus as *Pseudomonas* (mean count of nine) [46]. It is worth noting that our study found a slightly higher average BCG count per genome compared to a broad analysis across 68 phyla, where the average was three [47].

**Figure 2:**
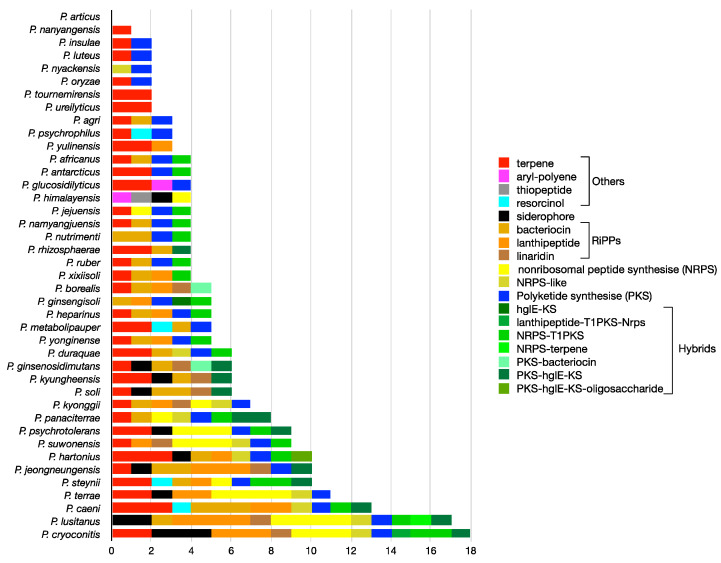
Occurrence of BGCs predicted using antiSMASH in the genomes of *Pedobacter* spp. according to their classes and sub-classes.

At least one BGC was found in all genomes (except *P. arcticus*), and *P. cryoconitis* and *P. lusitanus* have the highest number of clusters (18 and 17, respectively) (Figure 2; Table S1). There was no correlation between the genome size and the number of the BGCs per genome (Figure S1). The distribution of the BGCs across the genus, categorized by predicted product, displayed considerable heterogeneity (Figure 1). Nevertheless, terpene-related BGCs were prevalent in species that had only a single BGC per genome (Figure 1b).

### 3.2. The Pattern of Encoded SMs Is Phylogenetically Independent

The relationship between the distribution of biosynthetic gene clusters (BGCs) and phylogenetic lineage was examined, yielding an indiscernible pattern (Figure 1b). For instance, the species *P. lusitanus*, *P. cryoconitis*, *P. hartonius* and *P. himalayensis*, despite their close relationship, exhibited a distinct number and type of BGC arrays (Figure 1b and Figure 2). Focusing only on the total BGCs, *P. lusitanus*, *P. cryoconitis* and *P. caeni* are the species with the highest number, despite not being phylogenetically close (Figure 2). On the other hand, *P. ruber*, *P. namyangjuensis* and *P. africanus* occupy distant branches in the phylogenetic tree and encode the same type of SMs (Figure 1b and Figure 2). The same was observed for *P. nanyangensis* and *P. tournemirensis* that, despite being distantly related, both possess only one terpene BGC. Also, independently of the evolutionary similarities, almost all species have the potential to produce terpenes.

### 3.3. Most BGCs from Pedobacter spp. Do Not Share Homology with Each Other

**Figure 3:**
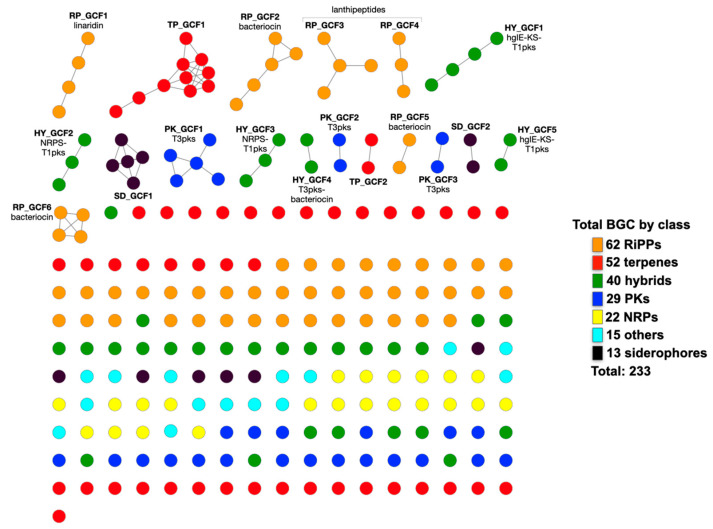
Overview of the sequence similarity network of 233 *Pedobacter* BGCs based on the BiG-SCAPE analysis output and visualized with Cytoscape [52]. Each node represents a BGC. On the right, the number of BGCs per class is provided. GCFs with more than one node were provided with a designation, as described in Table S2. The prefixes HY, PK, RP, SD and TP represent hybrid, PK, RiPPs, siderophore and terpene, respectively.

To identify the orthologous relationship of the BGCs, sequence similarity networks were generated based on the BiG-SCAPE analysis and visualized with Cytoscape (Figure 3). Out of the 233 BGCs identified, 169 were considered singletons and 64 were integrated in 18 undirected networks, making a total of 187 gene cluster families (GCFs; Figure 3; Tables S1 and S2). These results show that most BGCs from *Pedobacter* spp. show little or no homology with each other, and a significant proportion of the GCFs (73%) are unique to individual species (singletons). An exception is the largest identified GCF, which includes 10 BGCs that encode terpenes (Figure 3; Table S2). However, GCFs for other SMs consisting of at least two BGCs were also found, except for NRPs, all of which were singletons (Figure 3). The GCFs including only two clusters are derived primarily from closely related species (Table S2). Therefore, despite a general lack of correlation between the quantities and types of BGCs, certain species (phylogenetically related or not) might have the potential to produce identical or very similar SMs. We also found that only two species (*P. agri* and *P. ginsenosidimutans*) have all their BGCs integrated in GCF networks (Tables S1 and S2). On the contrary, *P. cryoconitis*, *P. lusitanus*, and *P. caeni* emerged as the species with highest count of GCFs as singletons, followed by *P. hartonius*, *P. psychrotolerans* and *P. steynii* (Table S1).

### 3.4. Most of the BGCs from Pedobacter spp. Should Encode the Production of New Compounds

The perspective of *Pedobacter* spp. producing novel SMs becomes apparent, as only 23% of their BGCs have any level of similarity to the BGCs found in the MIBiG repository, which is a reference database for BGCs with known functions [29]. The highest similarity identified (84%) was between the aryl polyene (APE) BGC of *P. himalayensis* and *E. coli* (Figure S2). APEs share structural and functional similarities with carotenoid pigments, despite their distinct biosynthetic pathways. Notably, both compounds contribute to protecting bacteria from oxidative stress [48,49].

Also, in *P. himalayensis*, a cluster encoding a nonribosomal peptide synthetase (NRPS), which are large enzymatic complexes engaged in the production of NRPs, showed a 57% similarity to the amonabactin NRPS of *A. hydrophila* ATCC 7966 (Table S1; Figure S2). Additionally, this cluster also includes enterobactin-related genes. Both amonabactin and enterobactin function as siderophores. Enterobactin is the strongest siderophore known, binding to the ferric ion (Fe^3+^) with a high affinity (Raines et al., 2016). We conducted an in silico analysis of this *P. himalayensis* NRPS and determined that its domains incorporate Ser and Dhb amino acids into the final peptide structure, which corresponds to enterobactin monomers [50]. Consequently, we hypothesize that *P. himalayensis* produces a enterobactin-like siderophore, probably in response to iron deficiency, as seen in *E. coli* (Hantash et al., 1997).

The third highest MIBiG similarity detected was between a terpene BGC of *P. oryzae* and a carotenoid BGC of *Algoriphagus* spp. (57%) [51]. Terpene BGCs of 22 other species, exhibited similarities to the same carotenoid BGC, although at percentages of less than 50% (Figure S2). This finding suggests that a considerable number of the terpene BGCs identified in this study may be linked to the biosynthesis of carotenoids, which are natural pigments that play diverse and essential roles in various biological systems. Next, eight siderophore BGCs showed a 50% similarity to a siderophore BGC responsible for the production of desferrioxamine E (Figure S2). These clusters encode enzymes belonging to the NRPS-independent siderophore (NIS) synthetases (conserved domain IucA/IucC) involved in the adenylation of a carboxylic acid substrate, typically citrate, or a derivative [35]. The BGCs encoding the production of these two types of SMs (siderophores and terpenes) were selected for an in-depth analysis, given their economic importance and promising biotechnological application, which will be discussed in more detail in the subsequent sections. The species selected for this analysis were *P. cryoconitis* and *P. lusitanus*, mainly due to their high number of BGCs, many of which did not yield matches with the MiBIG database. As these species are phylogenetically related to *P. hartonius* and *P. himalayensis*; these were also included in the analysis.

### 3.5. The Siderophore BGCs of P. cryoconitis and P. lusitanus Encode Bifunctional IucAC/AcD NIS Synthetases

**Figure 4:**
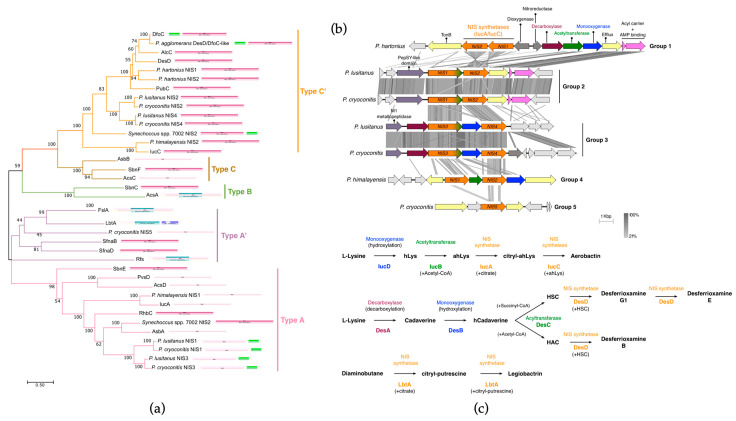
Neighbor-joining phylogenetic tree (**a**) of structurally characterized NIS synthetases and those found in the siderophore BGCs of *P. cryoconitis*, *P. lusitanus* and their close relatives [35]. The scale bar represents the number of amino acid substitutions per site. Tree branches were colored based on the NIS synthetases described by Carrol et al. (2018). Panel (**b**) presents the comparative genomic results of the siderophore BGCs via tBlastx analysis, where grey shading highlights regions of shared similarity, as indicated in the legend. Panel (**c**) offers a schematic representation of the biosynthetic pathway of known NISs, featuring some of the enzymes identified in the BGCs of *Pedobacter* spp.

A total of seven siderophore BGCs encoding the NIS biosynthetic pathway were identified in *P. cryoconitis*, *P. lusitanus* and their closely related strains (Figure 4b). Of these, three BGCs were from *P. cryoconitis*, two from *P. lusitanus,* and *P. himalayensis* and *P. hartonius* have one each (Figure 2; Table S1). In an attempt to simplify the description, we tried to categorize the BGCs based on their similarity (Figure 2) and identified that only two BGCs from *P. cryoconitis* and *P. lusitanus* showed similarity, and, therefore, were grouped together (Figure 4b). Considering the core biosynthetic enzymes, it was observed that each BGC, except for one from *P. cryoconitis* (Group 5 in Figure 4b), contained two NIS synthetases (Figure 4b). All the synthetases were subjected to phylogenetic analysis (Figure 4a), revealing that the *P. hartonius* cluster exclusively contains type C’ synthetases, whereas the two groups of *P. lusitanus* and *P. cryoconitis* (Group 2 and Group 3; Figure 4b), as well as the *P. himalayensis* BGC (Group 4; Figure 4b), harbor both type A and type C’ synthetases.

Classical enzymes of type A and type C’ include IucA and IucC, respectively. They play a pivotal role in the biosynthesis of aerobactin by sequentially coupling N^6^-acetyl-N^6^-hydroxylysine to the primary carboxylates of citrate (Figure 4c; [53]). Generally, type A enzymes are responsible for the condensation of citric acid or its derivatives with mono-amines or amides, forming the core structure of siderophores. They display a particular affinity for citric acid and less complex amine or amide substrates. Type C’ synthetases perform the condensation of these intricate building blocks and are often engaged in macrocyclization reactions, leading to the production of larger and more diverse siderophores [35]. The BGC of *P. himalayensis* (Group 4; Figure 4) was identified to possess both types, with NIS1 and NIS2 following in the same clade as IucA and IucC, respectively. On the other hand, the *P. hartonius* type C’ enzymes lacked the cognate type A enzymes and were in the same DfoC clade, despite not having an acetyltransferase (AcD) domain. DfoC-like synthetases feature an N-terminal AcD domain fused to the NIS domain (IucAC) and have been found in the genomes of Bacteroidota and Proteobacteria phyla [54,55]. Interestingly, the type A enzymes of Group 2 and Group 3 (NIS1 and NIS3 of *P. lusitanus* and *P. cryoconitis*) also possess these two domains, albeit in an inverse arrangement (N-terminal IucAC and a C-terminal AcD domains, herein referred to as IucAC/AcD). While this configuration of domains (IucAC/AcD) were previously detected in the siderophore BGCs of specific Cyanobacteria, namely, *Anabaena variabilis* and *Synechococcus* spp. [55], there are few studies focused on these synthetases so far. Finally, the NIS synthetase found in the G5 BGC of *P. cryoconitis* is classified as type A’ (NIS5; Figure 4a), which is a type A subgroup with unique enantioselective substrates [35].

#### *P. cryoconitis* and *P. lusitanus* May Produce a Mixture of Unknown Siderophores

The siderophore BGC of *P. himalayensis* (Group 4 cluster; Figure 4b) showed a 22% homology with aerobactin BGC from the MIBiG database (Figure S2) and, as previously mentioned, its NIS synthetases are affiliated with IucA and IucC. This cluster also encodes two other enzymes required for aerobactin production, namely an acetyltransferase and a monooxygenase (Figure 4b,c), suggesting that this species may produce aerobactin or a very similar siderophore.

The siderophore BGC found in *P. hartonius* (G1; Figure 4b) represents one of the previously mentioned clusters with similarity to desferrioxamine E BGC (Figure 4 and Figure S2). Apart from NIS synthetases, this cluster also contains genes encoding a decarboxylase, a monooxygenase and an acetyltransferase, which are the other enzymes required for desferrioxamine E production (Figure 4b,c). However, a closer analysis reveals additional genes encoding a dioxygenase and a nitroreductase (Figure 4b), suggesting that the siderophore produced by this species is distinct from desferrioxamine E after all. The two clusters of *P. lusitanus* and *P. cryoconitis* that form Group 3 also bear similarity to the desferrioxamine E BGC (Figure S2). These clusters feature a decarboxylase and a monooxygenase gene, while the bifunctional IucAC/AcD synthetase can account for the acetyltransferase function. Given the lack of characterized siderophores that are produced by these type of enzymes, it is likely that these clusters encode the production of a new type of siderophore instead of desferrioxamine E. Interestingly, similar BGCs were identified in the genomes of other *Pedobacter* spp., indicating that their products are widespread SMs within the genus (Figure S3).

The third siderophore BGC identified in *P. cryoconitis* (Group 5) contains an orphan type A’ NIS synthetase flanked by export-related genes (Figure 4b). A cluster with analogous proteins is responsible for the biosynthesis of legiobactin by *Legionella pneumophila* [56,57] (Figure 4c). However, without characterization, it remains uncertain whether *P. cryoconitis* can produce a compound similar to this siderophore.

In summary, *P. lusitanus* and *P. cryoconitis* seem to be the species that produce more siderophores (at least two each), justifying further investigation into their structure and function. Given the habitat of both species, they hold potential for applications in bioremediation and agriculture biotechnology. Siderophores can be used for the treatment of metal-contaminated samples or as plant growth promoters to increase crop yield in the context of global climate change [58,59,60,61].

### 3.6. P. cryoconitis and P. lusitanus Terpenes: Carotenoids and Squalene

**Figure 5:**
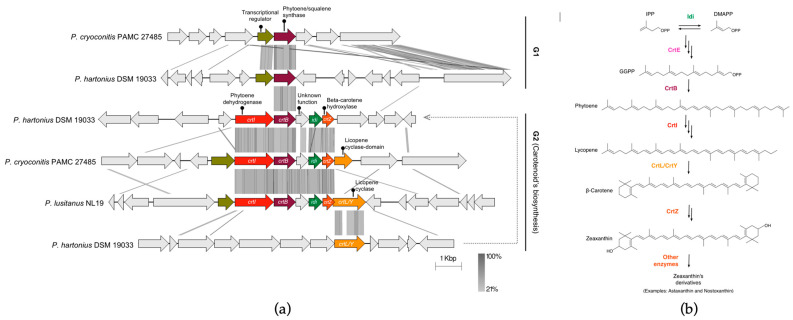
Comparative genomics of the six terpene BGCs found in *P. cryoconitis*, *P. lusitanus* and *P. hartonius* based on tBlastx analysis (**a**), where grey shadowing indicates regions of shared similarity according to the legend; (**b**) some biosynthetic pathways of squalene and known carotenoids, involving the production of beta-carotene, zeaxanthin and some derivatives.

In the *P. cryoconitis* and *P. lusitanus* related species group, a total of six BGCs encoding the biosynthesis of terpenes were identified (Figure 5a). In total, three BGCs were identified in *P. hartonius*, two in *P. cryoconitis*, one *in P. lusitanus* and none in *P. himalayensis*. The *P. lusitanus* cluster was classified via antiSMASH as a NRPS-terpene hybrid due to the presence of an NRPS within the adjacent 10 kb region of the terpene cluster. However, the association between these biosynthetic elements might not necessarily indicate the production of a hybrid compound. The comparative genomics analysis of the terpene BGCs resulted in their classification into Group 1 (G1) and Group 2 (G2) clusters (Figure 5a).

The common traits among G1 clusters comprise a phytoene/squalene synthase and a transcriptional regulator gene. Typically, in bacteria, these orphan synthases correspond to squalene synthases (SQSs; Figure 5b), which participate in the biosynthesis of squalene, a precursor of triterpenoids and steroids [62]. Squalene possesses notable properties, such as antioxidant, anticancer and anti-inflammatory [62,63]. Although various organisms from the three domains of life produce squalene, deep-sea shark liver oil has predominantly served as the natural source of this compound for several years [62]. Thus, due to the increased demand, sustainable alternatives are being sought, such as harnessing the biomass of microorganisms. Thus, *P. hartonius* and *P. cryoconitis* may have biotechnological potential in the production of bioactive squalene.

In addition to phytoene/squalene synthases, the G2 clusters also include other essential genes for the carotenoid biosynthetic pathway (Figure 5b), notably the lycopene cyclase (*crtL/Y*), the phytoene dehydrogenase (*crtI*), the beta-carotene hydroxylase (*crtZ*) and the isopentenyl pyrophosphate isomerase (*idi*) gene (Figure 5a). Thus, the phytoene/squalene genes of these clusters were classified as *crtB* that encode the phytoene synthase, which converts geranylgeranyl pyrophosphate (GGPP) molecules into phytoene, the precursor of carotenoid biosynthesis [64] (Figure 5b). In the case of *P. hartonius*, the carotenogenesis genes were identified as separate BGCs via antiSMASH, as the *crtL/Y* gene was not found in close proximity to the other genes (Figure 5a). Carotenoids are pigments, and as such, the G2 clusters are likely the biosynthetic clusters responsible for the synthesis of the light/sand yellow pigment observed in colonies of *P. lusitanus*, *P. cryoconitis* and *P. hartonius* [65,66,67]. The demand for carotenoids in cosmetics and human healthcare has steadily increased, prompting researchers and R&D companies to seek new alternatives [68,69,70]. Thus, these *Pedobacter* species and their BGCs deserve further investigation in this context.

## 4. Conclusions

The *Pedobacter* pangenome contains a substantial array of BGCs involved in the biosynthesis of compounds with promising biotechnological and commercial value, including RiPPS, terpenes, PKs, NRPs and siderophores. Additionally, some BGCs are expected to produce hybrid SMs. Most of the BGCs exhibit no significant similarities to their known counterparts in the MIBiG database, highlighting the biosynthetic diversity of *Pedobacter* spp. Our study revealed a plethora of SM clusters within the *Pedobacter* genomes, positioning this genus as a source of novel compounds ready for exploration, alongside other Gram-negative genera. Herein, *P. cryoconitis* and *P. lusitanus* emerged as excellent research models due to their abundant and diverse number of clusters that enable the production of various compounds such as siderophores, carotenoids and squalene, together with NRPs and lanthipeptides, all within a single strain. Given this profile, it would be interesting to harness the potential of these bacteria as plant probiotics, particularly considering the pressing challenge of promoting sustainable agriculture.

## Data Availability

Data herein analyzed is freely available in NCBI.

## Supplementary Materials

The following supporting information can be downloaded at https://www.mdpi.com/article/10.3390/microorganisms11102445/s1.
