# Supplementary material for: The Pangenome of Gram-Negative Environmental Bacteria Hides a Promising Biotechnological Potential
Source: Microorganisms. 2023 Sep 29;11(10):2445. doi: 10.3390/microorganisms11102445 (PMC10609062; doi:10.3390/microorganisms11102445)
Supplement: Supplementary file 1 [file microorganisms-11-02445-s001.zip › microorganisms-2622071-supplementary.pdf]

Supplementary Material

The pangenome of Gram-negative environmental bacteria hides a promising biotechnological potential.

Covas C, Figueiredo G, Gomes M, Santos T, Mendo S, Caetano T

Figures

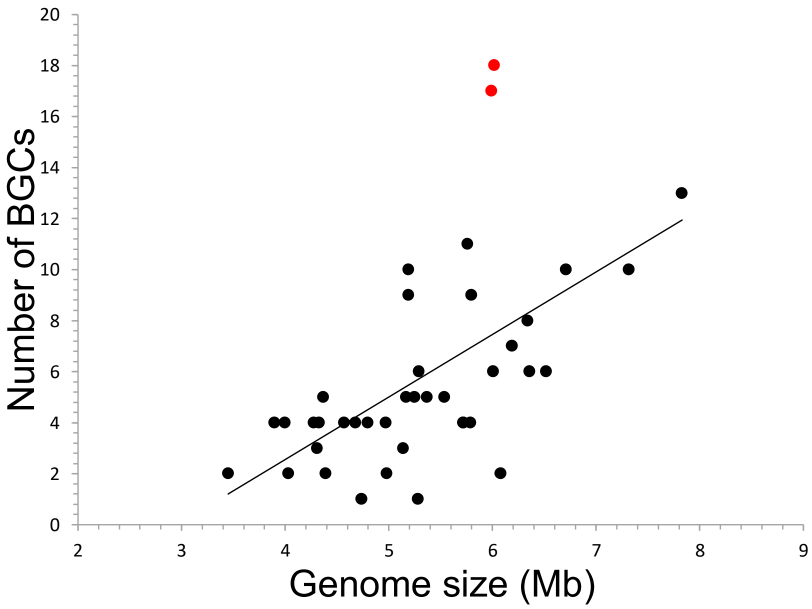

Figure S1: Relation between genome size of *Pedobacter* spp. and the number of BCGs found in each genome. The red points represent *P. cryoconitis* and *P. lusitanus* that are the strains with the highest number of BGCs.

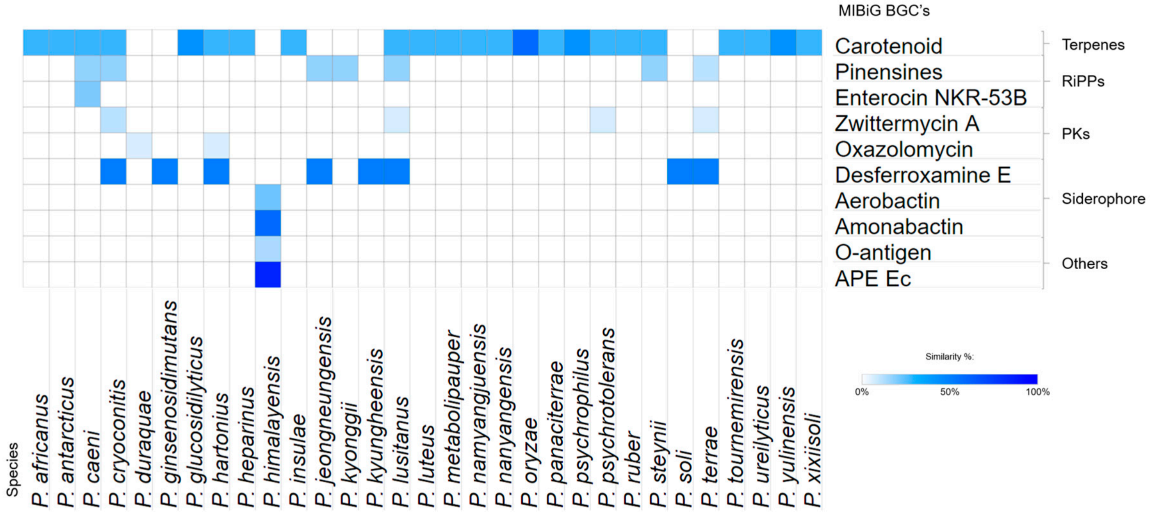

Figure S2: Heatmap of sequence similarity BGCs from *Pedobacter* genus. Columns represent the 31 *Pedobacter* species under study and the rows the MiBIG similarity hits and BGCs classes. The degree of similarity is represented by gradient color and was obtained from antiSMASH (Table S1).

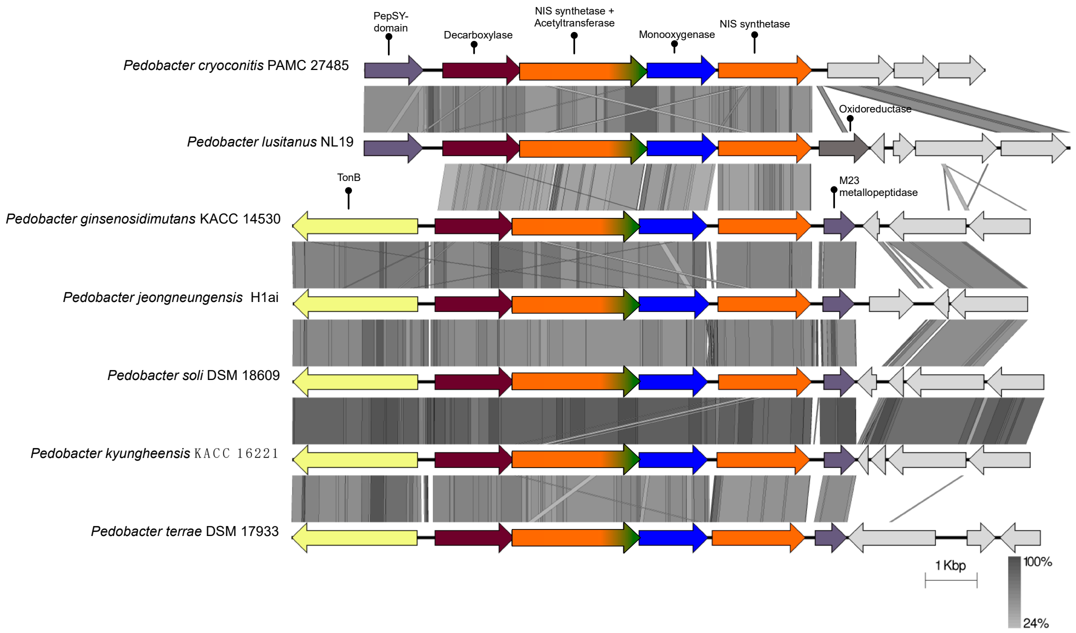

Figure S3: Genetic comparison between seven siderophore's gene clusters encoding the NIS synthase C-terminally fused to an acetyltransferase together with a decarboxylase and a monooxygenase identified in this study in *P. cryoconitis*, *P. lusitanus* and other *Pedobacter* species. Grey shadowing indicates regions of shared similarity according to the legend.

Table S1: Biosynthetic gene cluster identified in the *Pedobacter* species genome by antiSMASH and BiG-SCAPE tools. GCF: gene cluster family. GS: Genome size. GCF singleton and in network were written in black and red, respectively.

Clusters	BGC type	BGC class	GCF	Most similar known cluster	MIBiG BGC-ID	GS (Mb)
P. africanus DSM 12126T						5.72287
Cluster 1	Terpene	**Terpene**	FAM00122	Carotenoid_biosynthetic_gene_cluster (28% of genes show similarity)	BGC0000650_c1	
Cluster 2	Bacteriocin	**RiPPs**	FAM00123	-	-	
Cluster 3	T3pks	**PK**	FAM00124	-	-	
Cluster 4	NRPS-T1pks	**Hybrid**	FAM00125	-	-	
P. agri PB92T						5.14155
Cluster 1	Terpene	**Terpene**	FAM00030	-	-	
Cluster 2	Bacteriocin	**RiPPs**	FAM00184	-	-	
Cluster 3	T3pks	**PK**	FAM00185	-	-	
P. antarcticus 4BYT						4.56632
Cluster 1	T3pks	**PK**	FAM00009	-	-	
Cluster 2	NRPS-T1pks	**Hybrid**	FAM00010	-	-	
Cluster 3	Terpene	**Terpene**	FAM00011	-	-	
Cluster 4	Terpene	**Terpene**	FAM00012	Carotenoid_biosynthetic_gene_cluster (28% of genes show similarity)	BGC0000650_c1	
P. borealis DSM 19626T						5.54492
Cluster 1	T3pks-Bacteriocin	**Hybrid**	FAM00128	-	-	
Cluster 2	Lanthipeptide	**RiPPs**	FAM00129	-	-	
Cluster 3	Bacteriocin	**RiPPs**	FAM00130	-	-	
Cluster 4	Linaridin	**RiPPs**	FAM00070	-	-	
Cluster 5	Terpene	**Terpene**	FAM00030	-	-	
P. caeni DSM 16990T						7.82878
Cluster 1	Bacteriocin	**RiPPs**	FAM00107	-	-	
Cluster 2	Terpene	**Terpene**	FAM00108	-	-	
Cluster 3	Terpene	**Terpene**	FAM00109	Carotenoid_biosynthetic_gene_cluster (28% of genes show similarity)	BGC0000650_c	
Cluster 4	Resorcinol	**Others**	FAM00110	-	-	
Cluster 5	NRPS-T1pks	**Hybrid**	FAM00111	-	-	
Cluster 6	T1pks-hglE-KS	**Hybrid**	FAM00112	-	-	
		**RiPPs**		-	-	

Clusters	BGC type	BGC class	GCF	Most similar known cluster	MIBiG BGC-ID	GS (Mb)
Cluster 7	Lanthipeptide		FAM00113			
Cluster 8	Bacteriocin	**RiPPs**	FAM00114	-	-	
Cluster 9	T3pks	**PK**	FAM00115	-	-	
Cluster 10	Lanthipeptide	**RiPPs**	FAM00054	Pinensins_biosynthetic_gene_cluster (17% of genes show similarity)	BGC0001392_c	
Cluster 11	Terpene	**Terpene**	FAM00117	-	-	
Cluster 12	NRPS-like	**Others**	FAM00118	Enterocin_NKR-53B_biosynthetic_gene_cluster (20% of genes show similarity)	BGC0001291_c	
Cluster 13	Bacteriocin	**RiPPs**	FAM00119	-	-	
P. cryoconitis PAMC 27485T						6.01539
Cluster 1	T1pks-hglE-KS	**Hybrid**	FAM00037	-	-	
Cluster 2	NRPS-T1pks	**Hybrid**	FAM00038	-	-	
Cluster 3	Siderophore	**Siderophore**	FAM00039	Desferrioxamine_E_biosynthetic_gene_cluster (50% of genes show similarity)	BGC0001572_c1	
Cluster 4	NRPS-T1pks	**Hybrid**	FAM00040	Pinensins_biosynthetic_gene_cluster (17% of genes show similarity)	BGC0001392_c1	
Cluster 5	Terpene	**Terpene**	FAM00041	-	-	
Cluster 6	Lanthipeptide	**RiPPs**	FAM00042	-	-	
Cluster 7	Linaridin	**RiPPs**	FAM00043	-	-	
Cluster 8	Terpene	**Terpene**	FAM00044	Carotenoid_biosynthetic_gene_cluster (28% of genes show similarity)	BGC0000650_c1	
Cluster 9	NRPS-like	**Others**	FAM00045	-	-	
Cluster 10	NRPS	**NRP**	FAM00046	-	-	
Cluster 11	NRPS	**NRP**	FAM00047	-	-	
Cluster 12	T3pks	**PK**	FAM00048	-	-	
Cluster 13	Lanthipeptide-T1pks-NRPS	**Hybrid**	FAM00049	Zwittermycin_A_biosynthetic_gene_cluster (11% of genes show similarity)	BGC0001059_c1	
Cluster 14	Siderophore	**Siderophore**	FAM00050	-	-	
Cluster 15	NRPS	**NRP**	FAM00051	-	-	
Cluster 16	Siderophore	**Siderophore**	FAM00147	-	-	
Cluster 17	Lanthipeptide	**RiPPs**	FAM00053	-	-	
Cluster 18	Lanthipeptide	**RiPPs**	FAM00054	Pinensins_biosynthetic_gene_cluster (17% of genes show similarity)	BGC0001392_c1	
P. duraquae DSM 10034T					5.28826	
Cluster 1	NRPS-like	**Others**	FAM00217	-	-	
Cluster 2	Terpene	**Terpene**	FAM00218	-	-	

Clusters	BGC type	BGC class	GCF	Most similar known cluster	MIBiG BGC-ID	GS (Mb)
Cluster 3	T3pks	**PK**	FAM00219	-	-	
Cluster 4	Bacteriocin	**RiPPs**	FAM00220	-	-	
Cluster 5	Terpene	**Terpene**	FAM00221	-	-	
Cluster 6	NRPS-T1pks	**Hybrid**	FAM00222	Oxazolomycin_biosynthetic_gene_cluster (6% of genes show similarity)	BGC0001106_c1	
P. ginsengisoli T01R-27[T]					5.37336	
Cluster 1	Lanthipeptide	**RiPPs**	FAM00065	-	-	
Cluster 2	NRPS-T1pks	**Hybrid**	FAM00166	-	-	
Cluster 3	T3pks	**PK**	FAM00067	-	-	
Cluster 4	Bacteriocin	**RiPPs**	FAM00163	-	-	
Cluster 5	hglE-KS	**Hybrid**	FAM00069	-	-	
P. ginsenosidimutans KACC 14530[T]						6.51755
Cluster 1	Bacteriocin	**RiPPs**	FAM00028	-	-	
Cluster 2	Linaridin	**RiPPs**	FAM00070	-	-	
Cluster 3	T3pks-Bacteriocin	**Hybrid**	FAM00128	-	-	
Cluster 4	Siderophore	**siderophore**	FAM00029	Desferrioxamine_E_biosynthetic_gene_cluster (50% of genes show similarity)	BGC0001572_c1	
Cluster 5	T1pks-hglE-KS	**Hybrid**	FAM00207	-	-	
Cluster 6	Terpene	**Terpene**	FAM00030	-	-	
P. glucosidilyticus DD6b[T]						3.9019
Cluster 1	Arylpolyene	**Others**	FAM00133	-	-	
Cluster 2	Terpene	**Terpene**	FAM00134	Carotenoid_biosynthetic_gene_cluster (42% of genes show similarity)	BGC0000650_c1	
Cluster 3	Terpene	**Terpene**	FAM00135	-	-	
Cluster 4	T3pks	**PK**	FAM00136	-	-	
P. hartonius DSM 19033[T]						5.18872
Cluster 1	T3pks	**PK**	FAM00091	-	-	
Cluster 2	NRPS-like	**Others**	FAM00092	Oxazolomycin_biosynthetic_gene_cluster (6% of genes show similarity)	BGC0001106_c1	
Cluster 3	Siderophore	**siderophore**	FAM00093	Desferrioxamine_E_biosynthetic_gene_cluster (50% of genes show similarity)	BGC0001572_c1	
Cluster 4	Bacteriocin	**RiPPs**	FAM00094	-	-	
Cluster 5	Terpene	**Terpene**	FAM00095	-	-	
Cluster 6	Lanthipeptide	**RiPPs**	FAM00096	-	-	

Clusters	BGC type	BGC class	GCF	Most similar known cluster	MIBiG BGC-ID	GS (Mb)
Cluster 7	Terpene	**Terpene**	FAM00097	-	-	
Cluster 8	NRPS-T1pks	**Hybrid**	FAM00038	-	-	
Cluster 9	T1pks- hglE-KS Oligosaccharide	**Hybrid**	FAM00099	-	-	
Cluster 10	Terpene	**Terpene**	FAM00100	Carotenoid_biosynthetic_gene_cluster (28% of genes show similarity)	BGC0000650_c1	
P. heparinus DSM 2366^T						5.16945
Cluster 1	Lanthipeptide	**RiPPs**	FAM00017	-	-	
Cluster 2	T3pks	**PK**	FAM00018	-	-	
Cluster 3	NRPS-T1pks	**Hybrid**	FAM00019	-	-	
Cluster 4	Terpene	**Terpene**	FAM00020	Carotenoid_biosynthetic_gene_cluster (28% of genes show similarity)	BGC0000650_c1	
Cluster 5	Bacteriocin	**RiPPs**	FAM00021	-	-	
P. himalayensis HHS 22^T						4.67912
Cluster 1	Thiopeptide	**RiPPs**	FAM00013	O-antigen_biosynthetic_gene_cluster (14% of genes show similarity)	BGC0000781_c1	
Cluster 2	NRPS	**NRP**	FAM00014	Amonabactin P750 biosynthetic_gene_cluster (57% of genes show similarity)	BGC0001502_c1	
Cluster 3	Aryl polyene	**Others**	FAM00015	APE_Ec_biosynthetic_gene_cluster (84% of genes show similarity)	BGC0000836_c1	
Cluster 4	Siderophore	**siderophore**	FAM00016	Aerobactin_biosynthetic_gene_cluster (22% of genes show similarity)	BGC0000939_c1	
P. insulae DSM 18684^T						4.388
Cluster 1	Terpene	**Terpene**	FAM00105	Carotenoid_biosynthetic_gene_cluster (28% of genes show similarity)	BGC0000650_c1	
Cluster 2	T3pks	**PK**	FAM00106	-	-	
P. jejuensis TNB23^T					4.79581	
Cluster 1	T3pks	**PK**	FAM00196	-	-	
Cluster 2	NRPS	**NRP**	FAM00197	-	-	
Cluster 3	Terpene	**Terpene**	FAM00198	-	-	
Cluster 4	NRPS-T1pks	**Hybrid**	FAM00199	-	-	
P. jeongneungensis H1ai^T						6.70808

Clusters	BGC type	BGC class	GCF	Most similar known cluster	MIBiG BGC-ID	GS (Mb)
Cluster 1	Lanthipeptide	**RiPPs**	FAM00027	-	-	
Cluster 2	Bacteriocin	**RiPPs**	FAM00028	-	-	
Cluster 3	Siderophore	**siderophore**	FAM00029	Desferrioxamine_E_biosynthetic_gene_cluster (50% of genes show similarity)	BGC0001572_c1	
Cluster 4	Terpene	**Terpene**	FAM00030	-		
Cluster 5	Lanthipeptide	**RiPPs**	FAM00031	Pinensins_biosynthetic_gene_cluster (17% of genes show similarity)	BGC0001392_c1	
Cluster 6	Bacteriocin	**RiPPs**	FAM00032	-	-	
Cluster 7	T1pks-hglE-KS	**Hybrid**	FAM00033	-	-	
Cluster 8	T3pks	**PK**	FAM00185	-	-	
Cluster 9	Linaridin	**RiPPs**	FAM00035	-	-	
Cluster 10	Lanthipeptide	**RiPPs**	FAM00036	-	-	
P. Kyonggii K-4-11-1T					6.18618	
Cluster 1	Bacteriocin	**RiPPs**	FAM00028	-	-	
Cluster 2	NRPS	**NRP**	FAM00202	-	-	
Cluster 3	T3pks	**PK**	FAM00203	-	-	
Cluster 4	Terpene	**Terpene**	FAM00030	-	-	
Cluster 5	Lanthipeptide	**RiPPs**	FAM00031	Pinensins_biosynthetic_gene_cluster (17% of genes show similarity)	BGC0001392_c1	
Cluster 6	Linaridin	**RiPPs**	FAM00206	-	-	
Cluster 7	T1pks-hglE-KS	**Hybrid**	FAM00207	-	-	
P. kyungheensis KACC 16221T						6.35864
Cluster 1	Terpene	**Terpene**	FAM00137	-	-	
Cluster 2	Terpene	**Terpene**	FAM00030	-	-	
Cluster 3	Siderophore	**siderophore**	FAM00029	Desferrioxamine_E_biosynthetic_gene_cluster (50% of genes show similarity)	BGC0001572_c1	
Cluster 4	Bacteriocin	**RiPPs**	FAM00184	-	-	
Cluster 5	T1pks-hglE-KS	**Hybrid**	FAM00207	-	-	
Cluster 6	Linaridin	**RiPPs**	FAM00070	-	-	
P. lusitanus NL19T						5.98819
Cluster 1	Siderophore	**siderophore**	FAM00143	Desferrioxamine_E_biosynthetic_gene_cluster (50% of genes show similarity)	BGC0001572_c1	
Cluster 2	NRPS-T1pks	**Hybrid**	FAM00038	-	-	
Cluster 3	T1pks-hglE-KS	**Hybrid**	FAM00145	-	-	

Clusters	BGC type	BGC class	GCF	Most similar known cluster	MIBiG BGC-ID	GS (Mb)
Cluster 4	NRPS-Terpene	**Hybrid**	FAM00146	Carotenoid_biosynthetic_gene_cluster (28% of genes show similarity)	BGC0000650_c1	
Cluster 5	Siderophore	**siderophore**	FAM00147	-	-	
Cluster 6	T3pks	**PK**	FAM00048	-	-	
Cluster 7	Lanthipeptide	**RiPPs**	FAM00149	-	-	
Cluster 8	NRPS	**NRP**	FAM00150	-	-	
Cluster 9	Bacteriocin	**RiPPs**	FAM00151	-	-	
Cluster 10	NRPS	**NRP**	FAM00152	Pinensins_biosynthetic_gene_cluster (17% of genes show similarity)	BGC0001392_c1	
Cluster 11	NRPS	**NRP**	FAM00153	-	-	
Cluster 12	Lanthipeptide	**RiPPs**	FAM00154	-	-	
Cluster 13	Lanthipeptide	**RiPPs**	FAM00155	-	-	
Cluster 14	Linaridin	**RiPPs**	FAM00156	-	-	
Cluster 15	Lanthipeptide	**RiPPs**	FAM00054	Pinensins_biosynthetic_gene_cluster (17% of genes show similarity) (Caetano et al., 2020)	BGC0001392_c1	
Cluster 16	NRPS-like	**Others**	FAM00158	-	-	
Cluster 17	NRPS	**NRP**	FAM00159	Zwittermycin_A_biosynthetic_gene_cluster (7% of genes show similarity)	BGC0001059_c1	
P. luteus DSM 22385[T]						4.02864
Cluster 1	Terpene	**Terpene**	FAM00120	Carotenoid_biosynthetic_gene_cluster (28% of genes show similarity)	BGC0000650_c1	
Cluster 2	T3pks	**PK**	FAM00121	-	-	
P. metabolipauper DSM 19035 [T]					5.25114	
Cluster 1	Terpene	**Terpene**	FAM00223	-	-	
Cluster 2	Resorcinol	**Others**	FAM00224	-	-	
Cluster 3	Terpene	**Terpene**	FAM00225	Carotenoid_biosynthetic_gene_cluster (28% of genes show similarity)	BGC0000650_c1	
Cluster 4	Bacteriocin	**RiPPs**	FAM00184	-	-	
Cluster 5	T3pks	**PK**	FAM00227	-	-	
P. namyangjuensis 5G38[T]						4.33013
Cluster 1	NRPS-T1pks	**Hybrid**	FAM00192	-	-	
Cluster 2	Bacteriocin	**RiPPs**	FAM00184	-	-	
Cluster 3	Terpene	**Terpene**	FAM00194	Carotenoid_biosynthetic_gene_cluster (28% of genes show similarity)	BGC0000650_c1	

Clusters	BGC type	BGC class	GCF	Most similar known cluster	MIBiG BGC-ID	GS (Mb)
Cluster 4	T3pks	**PK**	FAM00195	-	-	
P. nanyangensis Q-4[T]						4.73626
Cluster 1	Terpene	**Terpene**	FAM00191	Carotenoid_biosynthetic_gene_cluster (28% of genes show similarity)	BGC0000650_c1	
P. nutrimenti DSM 27372[T]						5.71699
Cluster 1	T3pks	**PK**	FAM00187	-	-	
Cluster 2	NRPS-T1pks	**Hybrid**	FAM00188	-	-	
Cluster 3	Bacteriocin	**RiPPs**	FAM00189	-	-	
Cluster 4	Bacteriocin	**RiPPs**	FAM00190	-	-	
P. nyackensis DSM 19625[T]						6.07711
Cluster 1	NRPS-like	**Others**	FAM00126	-	-	
Cluster 2	T3pks	**PK**	FAM00127	-	-	
P. oryzae DSM 19973[T]						3.44577
Cluster 1	T3pks	**PK**	FAM00025	-	-	
Cluster 2	Terpene	**Terpene**	FAM00026	Carotenoid_biosynthetic_gene_cluster (57% of genes show similarity)	BGC0000650_c1	
P. panaciterrae O48[T]						6.3428
Cluster 1	Terpene	**Terpene**	FAM00160	Carotenoid_biosynthetic_gene_cluster (28% of genes show similarity)	BGC0000650_c1	
Cluster 2	T1pks-hglE-KS	**Hybrid**	FAM00161	-	-	
Cluster 3	T1pks-hglE-KS	**Hybrid**	FAM00161	-	-	
Cluster 4	Bacteriocin	**RiPPs**	FAM00163	-	-	
Cluster 5	NRPS-like	**Others**	FAM00164	-	-	
Cluster 6	T3pks	**PK**	FAM00067	-	-	
Cluster 7	NRPS-T1pks	**Hybrid**	FAM00166	-	-	
Cluster 8	NRPS	**NRP**	FAM00167	-	-	
P. psychrophilus CCM 8644[T]						3.99914
Cluster 1	Terpene	**Terpene**	FAM00174	Carotenoid_biosynthetic_gene_cluster (42% of genes show similarity)	BGC0000650_c1	
Cluster 2	Resorcinol	**Others**	FAM00175	-	-	

Clusters	BGC type	BGC class	GCF	Most similar known cluster	MIBiG BGC-ID	GS (Mb)
Cluster 3	T3pks	**PK**	FAM00176	-	-	
Cluster 4	Terpene	**Terpene**	FAM00177	-	-	
P. psychrotolerans DSM 103236^T					5.19395	
Cluster 1	T3pks	**PK**	FAM00208	-	-	
Cluster 2	Terpene	**Terpene**	FAM00209	Carotenoid_biosynthetic_gene_cluster (28% of genes show similarity)	BGC0000650_c1	
Cluster 3	T1pks-hglE-KS	**Hybrid**	FAM00210	-	-	
Cluster 4	NRPS	**NRP**	FAM00211	Zwittermycin_A_biosynthetic_gene_cluster (7% of genes show similarity)	BGC0001059_c1	
Cluster 5	Siderophore	**siderophore**	FAM00212	-	-	
Cluster 6	Terpene	**Terpene**	FAM00213	-	-	
Cluster 7	NRPS	**NRP**	FAM00214	Zwittermycin_A_biosynthetic_gene_cluster (7% of genes show similarity)	BGC0001059_c1	
Cluster 8	NRPS	**NRP**	FAM00215	-	-	
Cluster 9	NRPS-T1pks	**Hybrid**	FAM00216	-	-	
P. rhizosphaerae DSM 18610^T						5.7893
Cluster 1	Terpene	**Terpene**	FAM00101	-	-	
Cluster 2	Terpene	**Terpene**	FAM00102	-	-	
Cluster 3	T1pks-hglE-KS	**Hybrid**	FAM00103	-	-	
Cluster 4	Bacteriocin	**RiPPs**	FAM00104	-	-	
P. ruber DSM 24536^T						4.27928
Cluster 1	T3pks	**PK**	FAM00087	-	-	
Cluster 2	Bacteriocin	**RiPPs**	FAM00088	-	-	
Cluster 3	Terpene	**Terpene**	FAM00120	Carotenoid_biosynthetic_gene_cluster (28% of genes show similarity)	BGC0000650_c1	
Cluster 4	NRPS-T1pks	**Hybrid**	FAM00090	-	-	
P. steynii D14^T						7.32118
Cluster 1	T3pks	**PK**	FAM00055	-	-	
Cluster 2	Lanthipeptide	**RiPPs**	FAM00054	Pinensins_biosynthetic_gene_cluster (17% of genes show similarity)	BGC0001392_c1	
Cluster 3	Bacteriocin	**RiPPs**	FAM00057	-	-	
Cluster 4	T1pks-hglE-KS	**Hybrid**	FAM00058	-	-	
Cluster 5	NRPS-T1pks	**Hybrid**	FAM00059	-	-	
Cluster 6	Terpene	**Terpene**	FAM00060	-	-	

Clusters	BGC type	BGC class	GCF	Most similar known cluster	MIBiG BGC-ID	GS (Mb)
Cluster 7	Terpene	**Terpene**	FAM00061	Carotenoid_biosynthetic_gene_cluster (28% of genes show similarity)	BGC0000650_c1	
Cluster 8	Resorcinol	**Others**	FAM00062	-	-	
Cluster 9	NRPS	**NRP**	FAM00063	-	-	
Cluster 10	NRPS-T1pks	**Hybrid**	FAM00166	-	-	
P. soli DSM 18609^T						6.00642
Cluster 1	Linaridin	**RiPPs**	FAM00070	-	-	
Cluster 2	Terpene	**Terpene**	FAM00030	-	-	
Cluster 3	Bacteriocin	**RiPPs**	FAM00075	-	-	
Cluster 4	Siderophore	**Siderophore**	FAM00029	Desferrioxamine_E_biosynthetic_gene_cluster (50% of genes show similarity)	BGC0001572_c1	
Cluster 5	T1pks-hglE-KS	**Hybrid**	FAM00207	-	-	
Cluster 6	Bacteriocin	**RiPPs**	FAM00028	-	-	
P. suwonensis DSM 18130^T						5.80383
Cluster 1	Lanthipeptide	**RiPPs**	FAM00000	-	-	
Cluster 2	T3pks	**PK**	FAM00185	-	-	
Cluster 3	Linaridin	**RiPPs**	FAM00002	-	-	
Cluster 4	NRPS	**NRP**	FAM00003	-	-	
Cluster 5	Terpene	**Terpene**	FAM00030	-	-	
Cluster 6	NRPS-like	**Others**	FAM00005	-	-	
Cluster 7	NRPS-T1pks	**Hybrid**	FAM00006	-	-	
Cluster 8	NRPS	**NRP**	FAM00007	-	-	
Cluster 9	NRPS	**NRP**	FAM00008	-	-	
P. terrae DSM 17933^T						5.7551
Cluster 1	Lanthipeptide	**RiPPs**	FAM00031	Pinensins_biosynthetic_gene_cluster (11% of genes show similarity)	BGC0001392_c1	
Cluster 2	Terpene	**Terpene**	FAM00030	-	-	
Cluster 3	Siderophore	**siderophore**	FAM00029	Desferrioxamine_E_biosynthetic_gene_cluster (50% of genes show similarity)	BGC0001572_c1	
Cluster 4	Terpene	**Terpene**	FAM00079	-	-	
Cluster 5	T3pks	**PK**	FAM00185	-	-	
Cluster 6	Lanthipeptide	**RiPPs**	FAM00081	-	-	
Cluster 7	NRPS	**NRP**	FAM00082	Zwittermycin_A_biosynthetic_gene_cluster (7% of genes show similarity)	BGC0001059_c1	
Cluster 8	NRPS	**NRP**	FAM00083	-	-	

Clusters	BGC type	BGC class	GCF	Most similar known cluster	MIBiG BGC-ID	GS (Mb)
Cluster 9	NRPS	**NRP**	FAM00084	-	-	
Cluster 10	NRPS	**NRP**	FAM00085	-	-	
Cluster 11	NRPS-like	**Others**	FAM00086	-	-	
P. tournemirensis R1ᵀ					5.27684	
Cluster 1	Terpene	**Terpene**	FAM00200	Carotenoid_biosynthetic_gene_cluster (28% of genes show similarity)	BGC0000650_c1	
*P.ureilyticus*THGT11ᵀ					4.97619	
Cluster 1	Terpene	**Terpene**	FAM00228	-		
Cluster 2	Terpene	**Terpene**	FAM00229	Carotenoid_biosynthetic_gene_cluster (28% of genes show similarity)	BGC0000650_c1	
P. yonginense KCTC 22721ᵀ						4.37242
Cluster 1	Terpene	**Terpene**	FAM00030	-	-	
Cluster 2	NRPS-T1pks	**Hybrid**	FAM00183	-	-	
Cluster 3	Bacteriocin	**RiPPs**	FAM00184	-	-	
Cluster 4	T3pks	**PK**	FAM00185	-	-	
Cluster 5	Lanthipeptide	**RiPPs**	FAM00186	-	-	
P. yulinensis YL28-9ᵀ						4.31077
Cluster 1	Lanthipeptide	**RiPPs**	FAM00230	-	-	
Cluster 2	Terpene	**Terpene**	FAM00231	Carotenoid_biosynthetic_gene_cluster (42% of genes show similarity)	BGC0000650_c1	
Cluster 3	Terpene	**Terpene**	FAM00232	-	-	
P. xixiisoli CGMCC1.12803ᵀ						4.9747
Cluster 1	Terpene	**Terpene**	FAM00178	Carotenoid_biosynthetic_gene_cluster (28% of genes show similarity)	BGC0000650_c1	
Cluster 2	Lanthipeptide	**RiPPs**	FAM00179	-	-	
Cluster 3	NRPS-T1pks	**Hybrid**	FAM00180	-	-	
Cluster 4	Bacteriocin	**RiPPs**	FAM00181	-	-	

Table S2: Additional information regarding all the species that compose the GCFs shown in Error! Reference source not found.. The prefixes HY, PK, RP, SD and TP represent hybrid, PK, RiPPs, siderophore and terpene, respectively.

BGC class	Cytoscape networks	*Pedobacter* species	Product prediction	GCF
hybrids	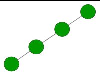	*P. soli*<br>*P. kyungheensis*<br>*P. kyonggii*<br>*P. ginsenosidimutans*	hglE-KS-T1pks	HY_GCF1<br>FAM00207
	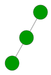	*P. hartonius*<br>*P. cryoconitis*<br>*P. lusitanus*	NRPS-T1pks	HY_GCF2<br>FAM00038
	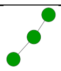	*P. ginsengisoli*<br>*P. panaciterrae*<br>*P. steynii*	NRPS-T1pks	HY_GCF3<br>FAM00166
	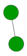	*P. ginsenosidimutans*<br>*P. borealis*	T3pks-Bacteriocin	HY_GCF4<br>FAM00128
	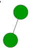	*P. panaciterrae*<br>*P. panaciterrae*	hglE-KS-T1pks	HY_GCF5<br>FAM00161
PKs	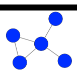	*P. jeongneungensis*<br>*P. suwonensis*<br>*P. yonginense*<br>*P. agri*<br>*P. terrae*	T3pks	PK_GCF1<br>FAM00185
	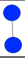	*P. ginsengisoli*<br>*P. panaciterrae*		PK_GCF2<br>FAM00067
	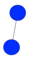	*P. cryoconitis*<br>*P. lusitanus*		PK_GCF3<br>FAM00048
RiPPs	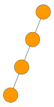	*P. soli*<br>*P. kyungheensis*<br>*P. borealis*<br>*P. ginsenosidimutans*	linaridin	RP_GCF1<br>FAM00070
	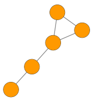	*P. agri*<br>*P. namyangjuensis*<br>*P. metabolipauper*<br>*P. yonginense*<br>*P. kyungheensis*	bacteriocin	RP_GCF2<br>FAM00184
	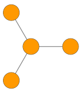	*P. caeni*<br>*P. cryoconitis*<br>*P. lusitanus*<br>*P. steynii*	lanthipeptide	RP_GCF3<br>FAM00054
	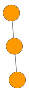	*P. terrae*<br>*P. jeongneungensis*<br>*P. kyonggii*	lanthipeptide	RP_GCF4<br>FAM00031
	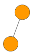	*P. ginsengisoli*<br>*P. panaciterrae*	bacteriocin	RP_GCF5<br>FAM00163
	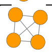	*P. kyonggii*<br>*P. soli*<br>*P. ginsenosidimutans*<br>*P. jeongneungensis*	bacteriocin	RP_GCF6<br>FAM00028
siderophores	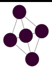	*P. ginsenosidimutans*<br>*P. jeongneungensis*<br>*P. kyungheensis*<br>*P. terrae*<br>*P. soli*	siderophore	SD_GCF1<br>FAM00029
	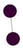	*P. cryoconitis*<br>*P. lusitanus*		SD_GCF2<br>FAM00147
terpenes	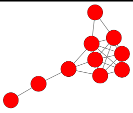	*P. suwonensis*<br>*P. terrae*<br>*P. jeongneungensis*<br>*P. ginsenosidimutans*<br>*P. kyonggii*<br>*P. yonginense*<br>*P. agri*<br>*P. borealis*	terpene	TP_GCF1<br>FAM00030

		P. soli<br>*P. kyungheensis*		
	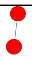	*P. ruber*<br>*P. luteus*	terpene	TP_GCF2<br>FAM00120
